# Altered Knee Joint Mechanics in Simple Compression Associated with Early Cartilage Degeneration

**DOI:** 10.1155/2013/862903

**Published:** 2013-01-29

**Authors:** Y. Dabiri, L. P. Li

**Affiliations:** Department of Mechanical and Manufacturing Engineering, University of Calgary, 2500 University Drive N.W., Calgary, AB, Canada T2N 1N4

## Abstract

The progression of osteoarthritis can be accompanied by depth-dependent changes in the properties of articular cartilage. The objective of the present study was to determine the subsequent alteration in the fluid pressurization in the human knee using a three-dimensional computer model. Only a small compression in the femur-tibia direction was applied to avoid numerical difficulties. The material model for articular cartilages and menisci included fluid, fibrillar and nonfibrillar matrices as distinct constituents. The knee model consisted of distal femur, femoral cartilage, menisci, tibial cartilage, and proximal tibia. Cartilage degeneration was modeled in the high load-bearing region of the medial condyle of the femur with reduced fibrillar and nonfibrillar elastic properties and increased hydraulic permeability. Three case studies were implemented to simulate (1) the onset of cartilage degeneration from the superficial zone, (2) the progression of cartilage degeneration to the middle zone, and (3) the progression of cartilage degeneration to the deep zone. As compared with a normal knee of the same compression, reduced fluid pressurization was observed in the degenerated knee. Furthermore, faster reduction in fluid pressure was observed with the onset of cartilage degeneration in the superficial zone and progression to the middle zone, as compared to progression to the deep zone. On the other hand, cartilage degeneration in any zone would reduce the fluid pressure in all three zones. The shear strains at the cartilage-bone interface were increased when cartilage degeneration was eventually advanced to the deep zone. The present study revealed, at the joint level, altered fluid pressurization and strains with the depth-wise cartilage degeneration. The results also indicated redistribution of stresses within the tissue and relocation of the loading between the tissue matrix and fluid pressure. These results may only be qualitatively interesting due to the small compression considered.

## 1. Introduction

Osteoarthritis (OA) is the most prevalent cause of disability among the elderly [1–3]. Among all joints, the knee has the highest incidence of OA [1, 4, 5]. The onset and progression of OA is related to the mechanical environment of articular cartilage [6]. In fact, the cartilage morphology, biosynthesis, and pathogenesis are strongly associated with its mechanical loading [7]. Therefore, the better the mechanical behavior of cartilage is understood, the better treatment and prevention strategies could be planned.

Osteoarthritis has been reported to initiate with deterioration from cartilage surface or cartilage-bone interface [8]. The former is believed to be a result of surface wear or splitting and the latter a result of high stiffness gradient at cartilage-bone interface [8, 9]. An altered mechanical environment, such as by stress, strain, and fluid flow, affects the biosynthesis of chondrocytes [10] and eventually leads to tissue degeneration and loss and exposure of bone surface to direct joint contact.

When OA is initiated from the surface, it progresses layer by layer from the superficial zone to the middle and eventually deep zones [11]. During this process, each layer of the tissue suffers from an altered mechanical environment; for example, the stress, strain, and fluid pressure in the deeper layer can be altered by degenerated superficial layers.

The mechanics of depth-wise (layer by layer) progression of OA in the knee joint must be affected by the multiple contacts between the cartilaginous tissues, including femoral cartilage, meniscus, and tibial cartilage. A few factors may be important in the contact mechanics of the knee. First, the 3D geometry of these tissues is obviously a dominant parameter that determines the contact area and distribution of contact loading. Second, the fluid pressurization in these tissues plays an essential role in the mechanical functions of the knee, because the knee compression is associated with high fluid pressure in these cartilaginous tissues [12]. Additionally, the depth-dependent tissue properties, often being characterized by three discrete zones, may also affect the mechanical behavior of the joint.

Great progress has been made in computational OA modeling, with major simplifications on the geometry including unrealistic boundary conditions and on the material properties including absence of fluid and fiber properties. For those studies with fluid pressure considered, some assumed a spherical contact in the knee with no meniscus [13–15]; others modeled unconfined compression testing only. The effect of PG depletion and collagen degradation was investigated by reducing the modulus of the two constituents, respectively [16]. Unconfined geometry was used with a fibril reinforced model [17]. In another study, OA was modeled in a depth-dependent manner [18]. The depth-dependent properties were used for cartilage based on values reported in the literature [19]. Again, unconfined compression geometry was used in the study. A major progress was made recently in knee OA modeling when both 3D geometry and fluid pressure in articular cartilage were implemented [20]. In this latest study the fluid flow in the menisci was ignored, which could possibly affect the prediction of the contact mechanics of the joint. Furthermore, the depth-dependent mechanical properties were not incorporated in the study.

Computer modeling may provide an effective tool to examine the effect of cartilage degeneration on contact mechanics and especially fluid pressure within the intact joint. We attempted to study the contact mechanics with an anatomically accurate finite element (FE) model of the normal and osteoarthritic knee joint. The material model for the cartilaginous tissues included nonfibrillar matrix, fibers, fluid, and depth-dependent properties. We hypothesized that, due to perturbations induced by OA, the fluid pressure in the tissue would be reduced with a given knee compression (displacement-control). To examine this hypothesis a normal model was compared with case studies whereby depth-wise progression of cartilage degeneration was implemented.

As a first step for our OA modeling of the knee, cartilage degeneration was assumed in the high load-bearing region of the medial condyle. This is one of the regions where the lesions are more likely progressed to deep layers [9, 21, 22], although OA lesions were also found in other sites of femoral cartilage [21, 23]. The medial condyle was chosen because it was believed to carry higher load compared to the lateral condyle [24]. The medial condyle was reported to be more susceptible to OA development in both normal [25] and ligament-deficient knees [26–28]. The medial condyle experienced the most rapid lesion progression [29].

## 2. Methods

**Figure 1:**
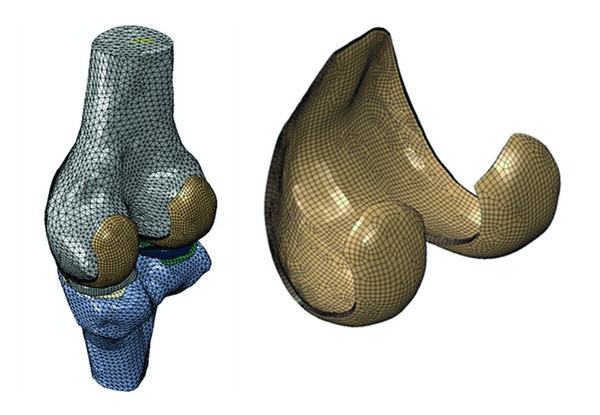
Finite element model of the tibiofemoral joint, showing the distal femur, proximal tibia, menisci, and femoral and tibial cartilages. The tibial cartilage on the medial side is essentially covered by the medial meniscus (right knee, medial side shown on the left of the figure). The femoral cartilage is further shown with 8 layers of elements.

The geometry of the model was reconstructed from MRI images of the right knee of a 27-year-old male subject, who had no symptoms of OA (SPGR sequence, 625 × 625 *μ*m resolution, sagittal scan). The model included the distal femur, femoral cartilage, meniscus, tibial cartilage, and proximal tibia (Figure 1). The maximum thickness of the femoral cartilage was approximately 2.8 mm, and the maximum thickness of the menisci was 8.4 mm [30].

The cartilaginous tissues, that is, femoral cartilage, menisci, and tibial cartilage, were assumed as fibril-reinforced fluid-saturated materials. A fibril-reinforced constitutive law was used which models the solid of the tissue as a linear nonfibrillar matrix that is reinforced by a nonlinear fibrillar matrix [17]. Hence, two material properties were required to define the nonfibrillar matrix, that is, the elastic modulus *E*_*m*_ and Poisson's ratio *ν*_*m*_. The fibrillar matrix was characterized by elastic moduli in three orthogonal directions. For the case of small deformations considered in the present study, these moduli were simplified as linear functions of the corresponding tensile strain, for example, for the local *x* direction

(1) $$\begin{array}{c} E_{x}=\{\begin{array}{cc} E_{x}^{0}+E_{x}^{\varepsilon }\varepsilon_{x}, & \text{if}\;\varepsilon_{x}\geq 0\\ 0, & \text{if}\;\varepsilon_{x}<0. \end{array} \end{array}$$

The compressive stiffness of the fibrillar matrix was neglected because the fibers mainly support tensile loading. Note that the *x* direction could be oriented in different directions for different sites. Therefore, a 3D collagen orientation could be thus incorporated. In order to describe the interstitial fluid flow, an orthotropic hydraulic permeability was introduced per Darcy's law, for example, for the local *x* direction

(2) $$\begin{array}{c} v_{x}=-k_{x}p_{f,x}, \end{array}$$

where *k*_*x*_ is the *x*-component of the permeability, which is the negative ratio of the *x*-component of the fluid velocity, *v*_*x*_, and the *x*-component of the fluid pressure gradient, *p*_*f*,*x*_. Simply replacing the subscript *x* in (1) and (2) with *y* or *z* would obtain the relevant equations for the *y* or *z* direction.

The depth-dependent properties were incorporated for the femoral cartilage; that is, the tissue properties varied with the superficial, middle, and deep zones, in the way approximated previously [31]. In the superficial zone, the fibers were oriented according to the split lines recorded from the surface ([32]; adopted from Figure 2 in [30]). In the middle zone, the fibers did not have any specific orientation. In the deep zone, they were vertical to the cartilage-bone interface. In the meniscus, the primary fibers were oriented in the circumferential direction. No preferred fiber directions were considered for the tibial cartilage due to lack of data.

The surface-to-surface contact was defined between articulating surfaces using ABAQUS 6.10. Namely, contact was defined between femoral cartilage and meniscus, femoral cartilage and tibial cartilage, and meniscus and tibial cartilage. No fluid flow was assumed between cartilage and bone. The cartilages and bones were meshed independently. However, in reality, the cartilage is firmly attached to the bone. There is no relative motion at the cartilage-bone interface. This interface condition was modeled using the TIE contact option provided by ABAQUS; that is, femoral cartilage was tied to femur, medial and lateral tibial cartilages were tied to tibia, and meniscus horns were tied to the tibial cartilage at both ends of each meniscus.

A ramp compression of 0.1 mm was applied in 1 s on top of the femur while the bottom of the tibia was fixed. The knee was in full extension. As a boundary condition, the free articulating surface (which was not in contact) was assigned to zero fluid pressure.

The consolidation procedure in ABAQUS was used to analyze the quasistatic problem. For cartilaginous tissues, porous elements with fluid pressure were used. The 20-node quadratic elements were used for the femoral cartilage, and the 8-node linear elements were used for tibial cartilage and meniscus. The choice of using different element types for the cartilages was a result of compromise between faster contact convergence and better fluid pressure distribution. The 20-node elements provide better numerical accuracy for the fluid pressure but significantly slow down the contact convergence. We used the 20-node elements for the femoral cartilage, because that was the focus for results. The bones were meshed with solid elements. The fluid pressure in the bones was not considered, because it is less significant in load support as compared to that in cartilaginous tissues due to a 3-order higher stiffness of the bones.

**Figure 2:**
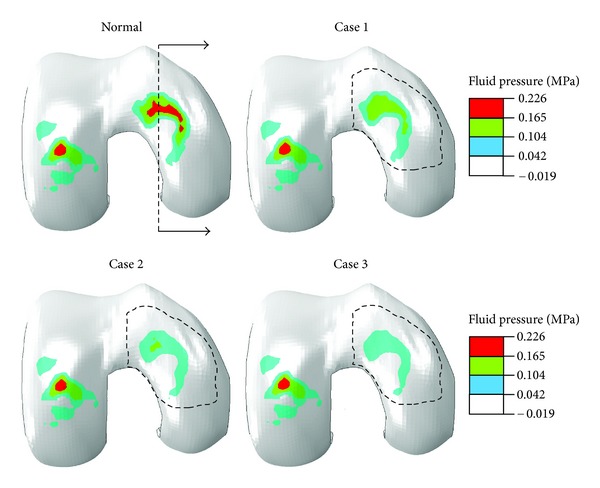
Fluid pressure (MPa) at the normalized depth of 1/16 (superficial layer) for the normal femoral cartilage and three cases of local cartilage degeneration. Case 1: degeneration in the superficial zone; Case 2: degeneration in both superficial and middle zones; and Case 3: degeneration in all three zones. The site of degeneration is indicated with the dash lines (inferior view of the right knee, i.e., the medial condyle on the right).

In order to understand the mechanics of the depth-wise progression of OA, the normal and three degenerative case studies were implemented computationally. In Case 1, the perturbations were implemented only in the superficial zone. In Case 2, the perturbations were implemented in superficial and middle zones, and in Case 3, the perturbations were implemented in all three zones. As discussed earlier, local cartilage degeneration was implemented within the high load-bearing region of the medial condyle of the femoral cartilage (Figure 2, bounded by the dash line). All other tissues were assumed normal. These three cases simulated the onset of cartilage degeneration from the superficial zone and progression to the deep zone.

The following perturbations were implemented for the degenerated cartilage: the permeability was increased by 50%, Young's modulus of fibrillar matrix was decreased by 70%, Young's modulus of nonfibrillar matrix was decreased by 65%, and the orientation of fibers was not set in any particular direction. The material properties of *normal* tissues are summarized in Table 1, which were mainly based on previous fibril-reinforced modeling with tissue explants [31, 33]. We assumed no changes in the thickness of the degenerated cartilage, because only early degeneration was considered. Therefore, the same tissue geometry was used for the normal and three case studies.

## 3. Results

**Figure 3:**
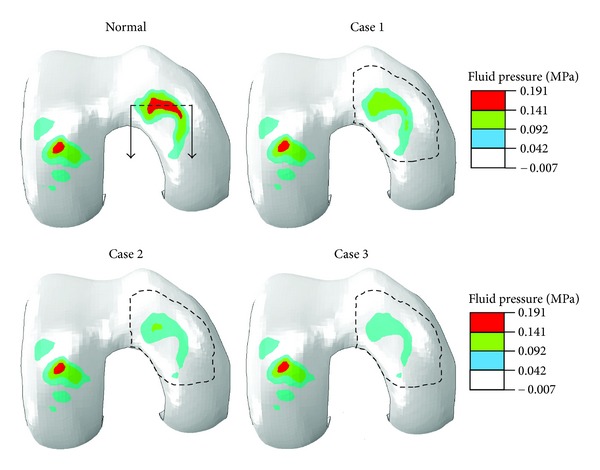
Fluid pressure (MPa) at the normalized depth of 13/16 (deep layer) for the normal femoral cartilage and three cases of local cartilage degeneration as defined in Figure 2.

All results presented here are for the end of ramp compression prior to relaxation. The fluid pressure in the femoral cartilage is shown in Figure 2 for a superficial layer and Figure 3 for a deep layer. In either layer, no significant alteration in the pressure was seen in the lateral condyle (left in figure) when cartilage degeneration advanced in the medial condyle from the superficial to middle and then deep zones (Normal → Case 1→ Case 2→ Case 3). The pore pressure in the medial condyle was substantially reduced with the progression of degeneration. This again was true for the fluid pressure in either superficial or deep layer.

**Figure 4:**
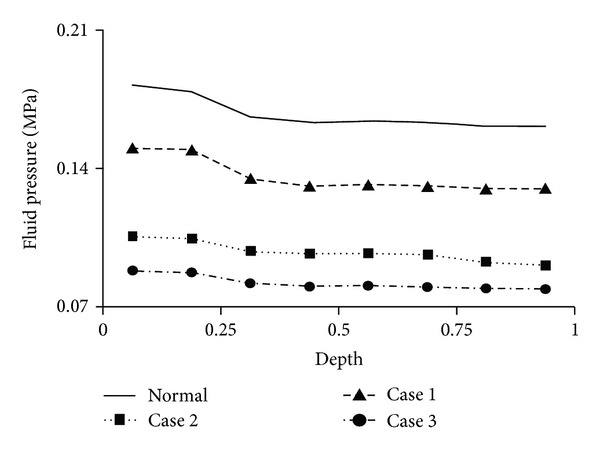
Variation of fluid pressure along the depth of the femoral cartilage, shown for a location in the central contact region of the medial condyle where cartilage degeneration occurred. The depth was normalized by the tissue thickness (0 = articulating surface; 1 = cartilage-bone interface). The pressure was calculated at the centroid of each element.

**Figure 5:**
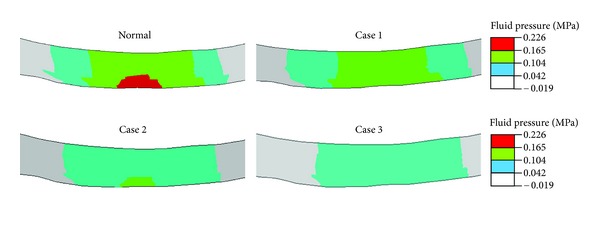
Fluid pressure (MPa) in a sagittal plane of the medial condyle (cut position shown in Figure 2) for the normal femoral cartilage and three cases of local cartilage degeneration as defined in Figure 2. The articulating surface is at the bottom, and the anterior side is on the right.

**Figure 6:**
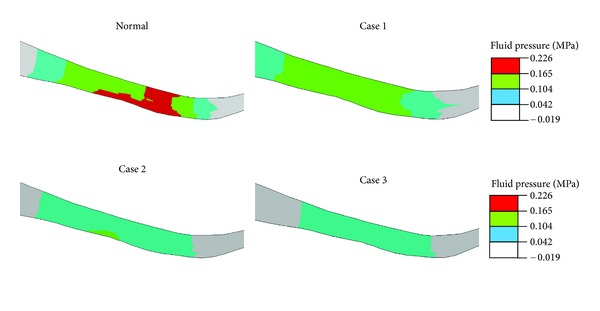
Fluid pressure (MPa) in a coronal plane of the medial condyle (cut position shown in Figure 3) for the normal femoral cartilage and three cases of local cartilage degeneration as defined in Figure 2. The articulating surface is at the bottom, and the lateral side is on the left.

The depth variation of the fluid pressure in the degeneration site is shown in Figures 4, 5, and 6. The pressure decreased with the tissue depth in all cases. However, the pressure gradient in the tissue thickness direction reduced progressively with cartilage degeneration for a given knee compression, with larger reduction in the superficial zone (Figure 4). The depth variation was also site-specific; it can be more easily seen in the high load-bearing region (Figures 5 and 6).

**Figure 7:**
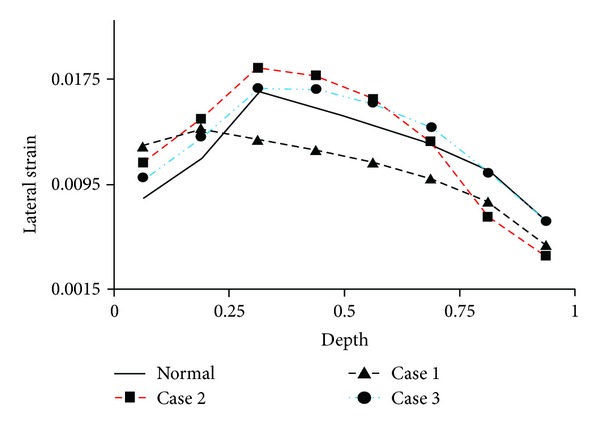
Lateral strain along the depth of the femoral cartilage, shown for a location in the central contact region of the medial condyle where cartilage degeneration occurred. This normal strain was in the direction parallel to the articulating surface and perpendicular to the split line. The depth was normalized by the tissue thickness (0 = articulating surface; 1 = cartilage-bone interface). The normal strain was calculated at the centroid of each element.

**Figure 8:**
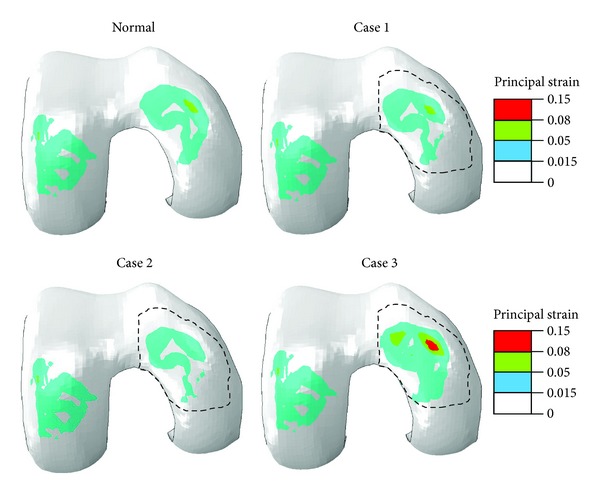
First principal strain at the normalized depth of 15/16 (deep layer) for the normal femoral cartilage and three cases of local cartilage degeneration as defined in Figure 2. This is an inferior view of the right knee; that is, the medial condyle is on the right.

The distribution of normal strain along the tissue depth was also altered with degeneration in the medial condyle (Figure 7). This strain was associated with the lateral expansion of the tissue when compressed in the thickness direction. The strain was smaller in the superficial zone because more tangentially oriented fibers there restrained the lateral expansion. However, the first principal strain was actually higher in the superficial zone than in the middle and most deep zones due to high shear strains at the surface (not shown). The first principal strain in the deepest layer was the largest in Case 3 (Figure 8), mostly because of large shear strains at the cartilage-bone interface in Case 3 (the lateral strain shown in Figure 7 was not the largest at the deepest layer).

**Figure 9:**
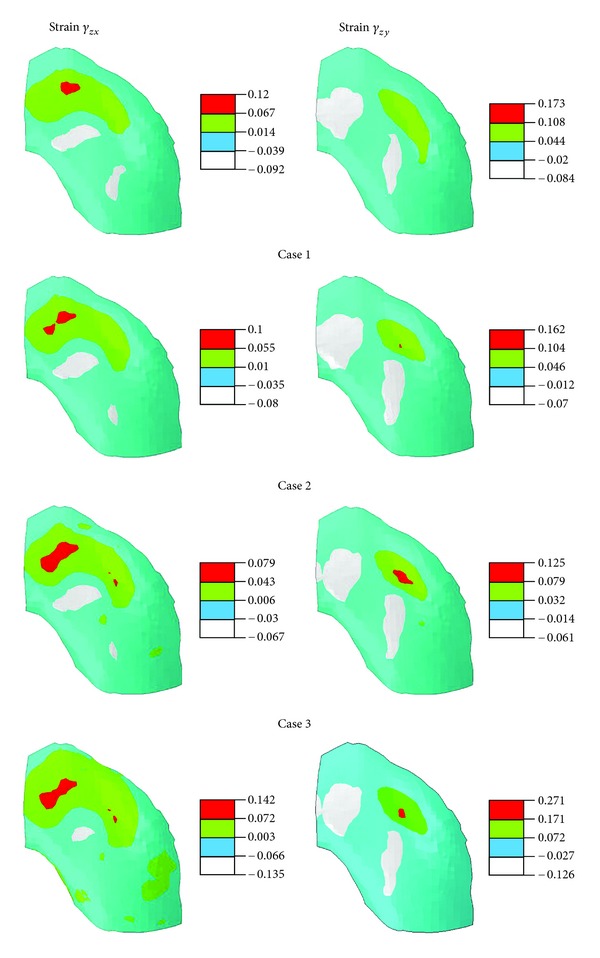
Shear strains at the normalized depth of 15/16 (deep layer) for the normal femoral cartilage and three cases of local cartilage degeneration as defined in Figure 2. This is shown for part of the medial condyle. The local *xy*-plane is parallel to the cartilage-bone interface. The corresponding shear stresses for the strains are *τ*_*zx*_ and *τ*_*zy*_, which are parallel to the interface.

As compared to the normal case, the shear strains at the cartilage-bone interface were reduced by cartilage degeneration in the superficial zone (Figure 9, Case 1 versus Normal) and further reduced when degeneration progressed into the middle zone (Figure 9, Case 2 versus Case 1). However, the shear strains were eventually raised above normal when cartilage degeneration progressed into the deep zone (Figure 9, Case 3 versus Normal). Note that these shear strains were associated with shear stresses *τ*_*zx*_ and *τ*_*zy*_, which might cause shear failure at the cartilage-bone interface (*z* is the tissue thickness direction).

## 4. Discussion

The fluid pressurization in all cartilaginous tissues was considered in the proposed model of cartilage degeneration in the human knee with anatomically accurate geometry of the joint. The zonal differences were considered in order to simulate the progression of degeneration from the superficial to deep zones. Our hypothesis was positively tested: for a given compression (displacement-control), the model predicted reduced fluid pressurization (Figures 2–6) although water content increased with cartilage degeneration. The fluid pressure can support a large portion of the load applied to cartilage [12], which is believed to be part of the mechanism to reduce the joint friction [34] and thus to reduce the chances of OA initiation from the tissue surface. Furthermore, the reduction in the fluid pressure observed in the present study for the case of displacement-control indicated increased joint friction and increased load support by the tissue matrix in the case of joint-force-control. Both may cause further progression of OA and deterioration of the tissue.

The onset of cartilage degeneration in an upper zone also resulted in reduced fluid pressure in the lower zone; for example, a degenerated superficial zone would reduce the fluid pressure in both middle and deep zones (Figure 4, Case 1). Since fluid pressurization bears high loading for the tissue, this result agrees with the protective role of the surface layer for the deep layer, as suggested by both experimental and computational studies [35, 36].

Furthermore, the fluid pressure reduced quickly when the degeneration started from the superficial zone and progressed to the middle zone, then reduced at a lower rate when the degeneration advanced to the deep zone (Figure 4). This was most likely a consequence of different fiber orientations in the three zones. In the superficial zone, the fibers are oriented tangentially to resist lateral expansion under knee compression and thus great fluid pressure is produced. Some tangential fibers in the middle zone should also contribute to increased fluid pressure. In the deep zone, however, the vertical fibers are in compression, and thus do not significantly contribute to fluid pressurization. Therefore, collagen degeneration in the deep zone would cause less fluid pressure change in the tissue than degeneration in the superficial and middle zones.

The shear strains at the cartilage-bone interface were increased substantially with cartilage degeneration to the deep zone (Figure 9, Case 3 versus Normal). This was probably because cartilage degeneration in the deep zone further increased the high gradient of the material properties from deep cartilage to underlying bone. Great shear strains at the cartilage-bone interface could cause microfractures, which eventually lead to OA [37–39]. The high gradients of material properties are believed to increase the possibility of damage to the cartilage-bone interface [8, 37]. Surprisingly, the shear strains at the interface were reduced in Cases 1 and 2 prior to the progression of degeneration into the deep zone (Figure 9, Case 1 or 2 versus Normal). The reason was probably due to the reduction of fluid pressure and its gradient in the tissue depth direction while the material properties in the deep zone remained unchanged in Cases 1 and 2. Note that knee compression was given in the present study (displacement-control). The shear strains might not have been reduced in Cases 1 and 2, if the joint force had been given (force-control).

Lower Young's moduli and higher permeability were used in the present study to simulate cartilage degeneration, in agreement with data from the literature [40, 41] The compressive modulus of cartilage was reduced, respectively, by 18% and 87.5%, and the water content was increased, respectively, by 79.9–81.6% and 84.1%, in moderate and advanced OA [41, 42]. According to another study, as a result of OA, the compressive and tensile moduli of human articular cartilage were decreased by 55–68% and 72–83%, respectively, and the permeability was increased by 60–80% [43]. For the human tibial cartilage, the compressive stiffness was decreased by 29% [44]; the compressive compliance was increased by 71% as a result of OA [45]. Six months after anterior cruciate ligament transection, the compressive modulus of canine cartilage was decreased by 25%, while the permeability was increased by ~48% twelve weeks after the surgery [46, 47]. We have used moderate values from these measurements.

Reduced surface fluid pressure with OA was also reported in the only similar existing study [20]. It was found in that study that the stress distribution through cartilage depth was also influenced by the orientation of superficial fibers. The additional features of the present study included the fluid pressure in all cartilaginous tissues and full consideration of the depth-dependent mechanical properties. We further simulated the depth-wise cartilage degeneration from the superficial to deep zones. As a consequence, the present results suggest that not only the degeneration in the superficial layer reduced the fluid pressure in the deeper layers, which agrees with the existing study [20], but also the degeneration in the deeper layers lowered the fluid pressure in the superficial layer.

A major limitation of the present study was due to the small knee compression (100 *μ*m) that was applied at a rather low rate (100 *μ*m/s) in the computer simulation. Our choice was a consequence of slow contact convergence and high demand in computational time resulting from a high resolution of element mesh associated with the zonal differences. Eight layers of elements were meshed in the tissue thickness direction so there were 2, 4, and 2 layers of elements, respectively, for the superficial, middle, and deep zones. This mesh required several times more computational time, as compared to the previous 4-layer mesh when the zonal differences were ignored [30, 48]. It took about a week to complete 1 s simulation on a 4 CPU workstation. In addition, we sometimes failed to obtain convergent results when larger or faster compressions were applied. Further verifications are in progress. Because of the small compression considered, one primary concern is whether the results were compromised by the geometrical errors introduced during MRI segmentation and element meshing, such as errors in surface curvature and tissue thickness. While such errors indeed existed, they were probably at a lower level as compared to 100 *μ*m. (The quality of surface construction can be positively seen from the continuous variation in pore pressure. The errors in geometry construction have been examined by independent research groups, e.g., [49].) Other limitations included the omission of osmotic pressure and the use of lab loading conditions.

The same compression was used in the present study; that is, a displacement-control was used for comparison. While the force-control loading protocol is often considered more realistic, a knee joint with different stages of OA may not experience the same force. As the OA develops, the patient tends to apply lower load on the diseased side [50]. On the other hand, it is more convenient and easier to interpret the results when using a displacement-control in both computer simulations and lab tests. Theoretically, the results from displacement-control can be qualitatively interpreted to that of force-control. Therefore, we chose the displacement-control for simplicity.

The results presented here should be qualitatively correct, although the magnitudes are not realistic because of the use of small and slow compression in the present study. The alterations due to degeneration would be amplified in the case of a physiologically realistic compression. This is because of the nonlinear and compression-rate dependent load response of the joint. If a larger compression was applied, the fluid pressure in the healthy cartilage would nonlinearly increase due to the normal collagen network in the tissue, while the pressure in the degenerated cartilage would increase more slowly due to a weak collagen network. For the same reason, if the same compression was applied faster, the fluid pressure would increase faster in the healthy cartilage than in the degenerated cartilage. In other words, the difference in the fluid pressurization in the healthy and degenerated cartilages would be enlarged with the compression magnitude and compression rate. This is understood from previous studies on cartilage explants: both nonlinearity and strain-rate dependence of the load response of cartilage are predominantly determined by the properties of collagen network [17, 51, 52].

 The results of this investigation shed light on the effect of perturbation of material properties and fibers orientation on knee joint mechanics, in the course of progression of OA from cartilage surface to the cartilage-bone interface. Clinical studies suggest the depth of cartilage defect as a parameter that characterizes OA severity [53]. Computational modeling can be used to study the effect of this parameter on the mechanics of knee joint. Furthermore, the role of defect depth in knee joint mechanics can be better understood if computational models consider depth-dependent properties embedded in an anatomical accurate geometry, as this study showed. The findings of this study could be implemented in characterizing OA severity based on the depth of cartilage injury. In fact, the development of OA is a multifactorial phenomenon including alteration of tissue mechanical properties, perturbation of fiber orientation, cartilage tissue loss, and the size and location of cartilage lesion [20, 53–55]. In this study, the effect of the first two parameters was investigated whereas the importance of other factors will be investigated in future. 

In summary, we have determined the alterations of fluid pressure and strains in articular cartilage for the local tissue degeneration in the medial condyle of the femur. These results may provide new information in understanding the progression of osteoarthritis. As discussed earlier, cartilage degeneration resulted in reduced capability of fluid pressurization and reduced pressure gradients in the tissue, which suggest reduced lubrication in the joint and increased load support for the tissue matrix. Results also suggest that once cartilage degeneration is initiated from the articulating surface, it will eventually advance to the deep layer. This facilitation is achieved through the reduction of fluid pressurization in all three zones with greater reduction in the superficial zone and damage to the depth-dependent structure of the tissue. In particular, cartilage degeneration in the superficial zone may increase the possibility of damage to cartilage-bone interface.

## Figures and Tables

**Table 1:** Material properties for the normal tissues (modulus: MPa; permeability: 10^−3^ mm^4^/Ns). The *x* is the primary fiber direction, that is, the split-line direction for the superficial zone, the depth direction for the deep zone for articular cartilage, and the circumferential direction for the meniscus. The *y* and *z* are normal to the primary fiber direction. The properties are the same in the *y* and *z* directions.

Tissue	Fibrillar matrix, (1)		Nonfibrillar matrix		Permeability, (2)	
	*E*_*x*_	*E*_*y*_ or *E*_*z*_	*E*_*m*_	*υ*_*m*_	*k*_*x*_	*k*_*y*_ or *k*_*z*_
Femoral cartilage						
Deep zone	3 + 1600*ε*_*x*_	0.9 + 480*ε*_*y*/*z*_	0.80	0.36	1.0	0.5
Middle zone	2 + 1000*ε*_*x*_	2 + 1000*ε*_*y*/*z*_	0.60	0.30	3.0	1.0
Superficial zone	4 + 2200*ε*_*x*_	1.2 + 660*ε*_*y*/*z*_	0.20	0.16	1.0	0.5
Tibial cartilage	2 + 1000*ε*_*x*_	2 + 1000*ε*_*y*/*z*_	0.26	0.36	2.0	1.0
Menisci	28	5	0.50	0.36	2.0	1.0
Bones	*E* = 5000		*υ* = 0.30			
